# Respiratory Trajectory after Invasive Interventions for Patent Ductus Arteriosus of Preterm Infants

**DOI:** 10.3390/children8050398

**Published:** 2021-05-15

**Authors:** Yu-Jen Wei, Yen-Ju Chen, Yung-Chieh Lin, Chung-Dann Kan, Min-Ling Hsieh, Yuh-Jyh Lin, Jing-Ming Wu, Jieh-Neng Wang

**Affiliations:** 1 Department of Pediatrics, National Cheng Kung University Hospital, College of Medicine, National Cheng Kung University, Tainan 70403, Taiwan; garlicwei@hotmail.com (Y.-J.W.); yensweet@gmail.com (Y.-J.C.); drapple@mail.ncku.edu.tw (Y.-C.L.); mactep_8@hotmail.com (M.-L.H.); ped1@mail.ncku.edu.tw (Y.-J.L.); jingming@mail.ncku.edu.tw (J.-M.W.); 2 Department of Surgery, National Cheng Kung University Hospital, College of Medicine, National Cheng Kung University, Tainan 70403, Taiwan; kcd56@mail.ncku.edu.tw

**Keywords:** patent ductus arteriosus, prematurity, transcatheter, ligation, respiratory trajectory

## Abstract

Invasive interventions have been conducted in preterm infants with significant patent ductus arteriosus (PDA) when medical treatment has failed, and methods of invasive intervention have been reported. Surgical ligation via lateral thoracotomy has been a well-established procedure for decades. Recently, transcatheter occlusion has been safely and feasibly applied to the premature population. However, little research has been conducted on the benefits of transcatheter occlusion in very-low-birth-weight (VLBW) infants compared to surgical ligation. This study compared transcatheter and surgical techniques in VLBW infants in terms of short-term respiratory outcomes. The medical records of 401 VLBW infants admitted to a tertiary hospital between September 2014 and January 2019 were retrospectively reviewed. Patients who were diagnosed with a congenital anomaly, a chromosomal anomaly, or congenital heart disease, except for an inter-atrial shunt, were excluded. The perinatal conditions, neonatal morbidities, periprocedural vital signs, and respiratory support trajectories were compared between the transcatheter-treated and surgically ligated group. A total of 31 eligible VLBW infants received invasive intervention: 14 were treated with transcatheter occlusion (Group A), and 17 infants were treated with surgical ligation (Group B). Respiratory outcomes were not statistically significant between the two groups, despite Group A showing a trend toward early improvement in post-intervention respiratory trajectory. In this small case study, a different trend in post-intervention respiratory trajectories was observed. Future research with larger case numbers should be conducted to address our preliminary observations in more detail.

## 1. Introduction

The ductus arteriosus is a vessel between the pulmonary circulation and systemic circulation in a fetus [1]. In most term infants, the ductus arteriosus closes spontaneously [2]. However, the spontaneous closure rate is inversely related to gestational age in premature infants, ranging from 13% to 98% [3]. Patent ductus arteriosus (PDA) among premature infants plays a role in hemodynamic instability and may be responsible for prematurity-related complications [4,5]. Persistent PDA has been associated with prematurity-related complications, such as pulmonary hemorrhage [6,7], chronic lung disease [8,9], intraventricular hemorrhage [10,11,12], renal dysfunction, necrotizing enterocolitis [13], neurological impairments [14], and mortality [5,15].

Supportive therapy or medical therapy is the initial strategy for PDA management [16]. However, invasive interventions have been required for hemodynamic-significant PDA (HS-PDA) when medical treatment has failed [17,18]. To date, invasive interventions for PDA have included surgical ligation or transcatheter occlusion. Surgical ligation for PDA in preterm infants was first reported in 1970 [19,20], and it has been well developed and reported to reduce mortality in extremely premature infants [21]. Surgical ligation has been a well-established procedure for decades [17,22,23]. However, re-expansion pulmonary edema [24], decreased lung compliance, vocal cord paralysis, and diaphragmatic paralysis after surgical ligation have all been reported [25,26,27,28].

Recently, transcatheter occlusion for PDA has established itself as a method used in term infants, but it has also recently been applied to the premature population [29,30,31]. Research has shown no difference in success rates between transcatheter occlusion and surgical ligation [31]; however, earlier respiratory improvements have been scantly reported for transcatheter occlusion compared to surgical ligation [32,33]. The pulmonary benefits of transcatheter occlusion deserve more research attention.

The authors of this study have previously reported the feasibility of transcatheter occlusion for very-low-birth-weight (VLBW, birth bodyweight ≤ 1500 g) infants of this study unit [34]. This current study was conducted to investigate the short-term respiratory outcome of preterm infants who received transcatheter occlusion or surgical ligation.

## 2. Materials and Methods

### 2.1. Study Design

The study aimed to identify the differences in the short-term respiratory outcomes of VLBW infants between two invasive interventions for PDA in VLBW infants: surgical ligation and transcatheter occlusion. The medical records of VLBW infants admitted to the National Cheng Kung University Hospital from September 2014 to January 2019 were retrospectively reviewed. VLBW infants who received invasive intervention, surgical ligation, or transcatheter occlusion for PDA were included. Infants with a congenital anomaly, a chromosomal anomaly, or congenital heart disease, except for an inter-atrial shunt, were excluded.

Since 2014, a group of interventionalists based at this institution have offered an alternative choice to surgical ligation for HS-PDA. While HS-PDA is oftentimes treated by means of a surgical ligation, a shared decision-making process were offered to the infant’s family. The procedure and potential risks were clearly explained by an interventionalist as well as by a surgeon. Following the family’s choice regarding surgical ligation or transcatheter occlusion, informed consent was obtained for the procedure.

After reviewing their medical records, the infants were divided into a transcatheter occlusion group (Group A) and a surgical ligation group (Group B). The study protocol was approved by our Institutional Review Board (protocol code A-ER-109-137, date: 30 April 2020).

### 2.2. Study Setting

This study was conducted in a 20-bed tertiary neonatal intensive care unit (NICU) at the National Cheng Kung University Hospital in Tainan, Taiwan. The care volume of this unit is approximately 350–400 neonates, treated yearly, including approximately 70–80 VLBW infants. Admitted infants are regularly cared for by two neonatologists, two residents, and one nurse practitioner.

### 2.3. Variable Collection or Definition

#### 2.3.1. Clinical Variables

The basic demographic data comprise sex, gestational age, and birth bodyweight. Age, body weight, medical condition, and respiratory data before and after the procedure day were recorded. Respiratory data included a fraction of oxygen (FiO_2_), mean airway pressure (for positive ventilation), and mode of ventilator settings.

#### 2.3.2. Definition of HS-PDA and the Procedure of Interventions

Invasive interventions for PDA in VLBW infants were decided when the hemodynamic significance of PDA persisted after the failure or contraindication of medical therapies. Criteria for determining a hemodynamically significant PDA were as follows: (1) a cardiovascular disease score ≥ 3 [35], (2) a pulmonary edema diagnosed on a plain chest film, (3) a pulmonary hemorrhage, (4) an enlarged left atrium with a left atrium to aortic root ratio (LA/Ao ratio) greater than 1.3, and (5) increased pulmonary venous flow.

Surgical ligation was regularly performed in the NICU of the study setting. Infants were intubated, sedated, treated to control pain, and closely monitored for vital signs by anesthesiologists. Continuously monitored vital signs included heart rate, respiratory rate, blood pressure, and oxygen saturation.

Transcatheter occlusion was performed in two steps, as described in our previous report [34]. First, we performed either femoral or umbilical venous cannulation with a 4 Fr sheath in the NICU on a warmer to prevent excessive heat loss, with close monitoring via intensive care monitors. Infants were lightly sedated with midazolam, and pain control was managed with fentanyl. For infants who breathed room air or used a nasal cannula, we applied continuous positive airway pressure (CPAP) on them during the procedure because of their sedated state, and we also ensured that they could tolerate the ventilation strategy under monitoring; for other infants, the ventilation strategy maintained was the same. Second, after vascular access was established, the infant, together with the warmer, was transferred to the catheterization laboratory and transcatheter treatment began, as the authors previously reported [34].

#### 2.3.3. Primary Outcome: Post-Intervention Respiratory Trajectory

The pulmonary score was set to represent respiratory trajectory as the reference [36]. A pulmonary score was calculated for every infant before their procedure (Day 1), on the procedure day (Day 0), and every 24 h after the procedure (Day 1–Day 10) (12 days in total). This score summarized a weighted sum of respiratory support and medical therapies. A higher score represented higher respiratory support. Changes in the two groups were compared from Day 1 to Day 10 (12 days in total).

Successful weaning from mechanical ventilation was defined as spontaneous breathing without a ventilator for three consecutive days. Weaning from oxygen support was defined as successful maintenance of oxygen saturation at more than 90% under room air for three consecutive days.

### 2.4. Statistic Analysis

Categorical data were reported as proportions or percentages. Continuous variables were reported as means ± standard deviations or median (interquartile range). Categorical variables were tested using the chi-square test or Fisher’s exact test for proportions. Continuous variables were tested using the Shapiro–Wilk test for normal distribution and *t*-test to compare means if the variables were normally distributed, and a Mann–Whitney U test was used if they were not. Statistical significance was set at *p* < 0.05.

## 3. Results

**Figure 1:**
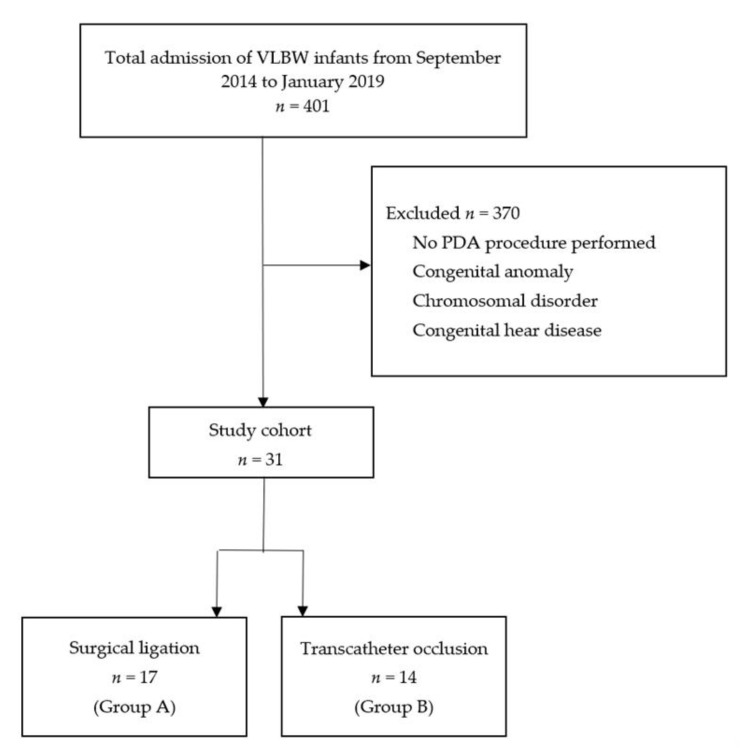
Algorithm for study population enrollment.

The medical charts of 401 VLBW infants were reviewed, and 31 eligible preterm infants were found to undergo PDA intervention during this period (Figure 1). Of the 401 VLBW infants, only 147 had ever received therapeutic medication for significant PDA. Among these enrolled infants, 14 underwent transcatheter occlusion (Group A) and 17 received surgical ligation (Group B).

Table 1 shows a comparison of the demographic data and ventilatory status of the two groups of infants 24 h before the procedure. Birth gestational age, age at PDA invasive intervention, vital signs, and baseline respiratory conditions were not different between the two groups. Comparisons between the two groups revealed no differences in FiO_2_, mean airway pressure for positive ventilation, positive end-expiratory pressure (PEEP), or pre-procedural intubation. Importantly, the baselines of the pulmonary scores 24 h before the procedure were similar between the two groups (*p* = 0.705).

The detailed reasons that VLBW infants required invasive intervention are shown in Table A1.

Table 2 shows the other respiratory outcome of infants after their invasive interventions. There was a shorter trend of post-procedural oxygen-dependent days in Group A compared with Group B (*p* = 0.083). Moreover, days with any positive pressure ventilator (including CPAP, invasive, or non-invasive positive pressure ventilation) for infants after transcatheter closure were significantly shorter than for infants undergoing surgical ligation (*p* = 0.033).

**Figure 2:**
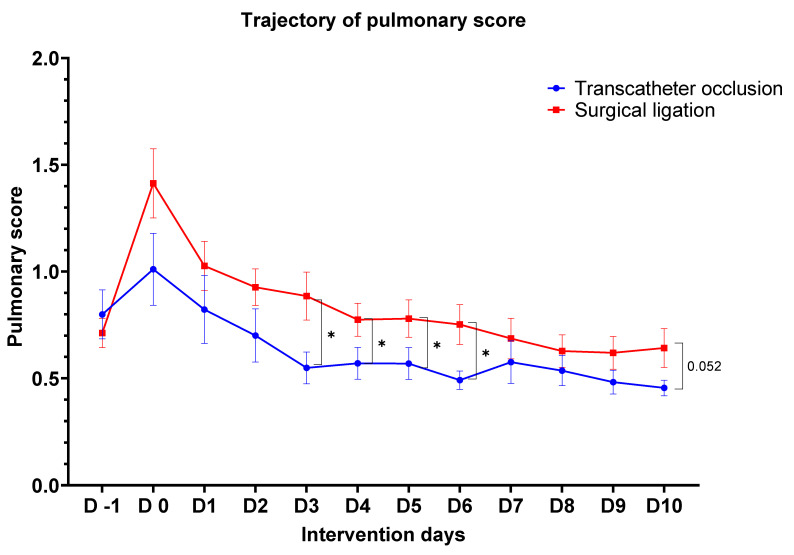
Trajectory of pulmonary scores before and after intervention. Data are presented as the mean ± standard error of the mean. Detailed data are listed in Table A2. * *p* < 0.05.

Figure 2 shows the respiratory trajectories of pulmonary scores after invasive intervention. One infant in Group A died on Day 1 because of intrauterine infection-related sepsis; the other 30 infants of this cohort survived during the 12-day observational period. Pulmonary scores were universally elevated on procedure day (Day 0) compared with pre-procedural day (Day 1). However, respiratory trajectory after Day 1 showed particularly interesting patterns. In Group A, the pulmonary score returned below its pre-procedural value on post-procedural day 2 (Day 2). In Group B, the pulmonary score remained elevated above its pre-procedure value until Day 7, despite a declining slope in both groups.

From Days 3 to 6, infants in Group A had a significantly lower pulmonary score than infants in Group B (all *p*-values < 0.05, respectively). On Day 10, a tendency of difference between the two groups was still observed (*p* = 0.052). The detailed daily pulmonary scores of the two groups are listed in Table A2.

Table A3 shows the hemodynamics of pre- and post-closure in the two groups, including systemic BP values and vasopressors equivalents [37]. Table A4 shows incidences of prematurity-related comorbidities at discharge in the two groups. No statistical differences were observed between the two groups in Table A3 and Table A4.

## 4. Discussion

In this study, we reported our preliminary observations for trajectories of pulmonary scores before and after interventional PDA closure in VLBW infants. With limited case numbers based on similar initial conditions, this study observed a tendency toward an early improvement of the pulmonary score in the transcatheter occlusion group of infants compared to the surgical ligation group. An in-depth analysis of the possible mechanism of our observation may not be within the scope of this article; however, several of our findings are worthy of consideration.

### 4.1. Comparision of Invasive Techniques in Closing PDA in VLBW Infants

In the early 2000s, the feasibility of cardiac catheterization of low-birth-weight infants was reported [38]. Later, the same was reported in very preterm infants [39]. The respective techniques and equipment have also been well developed, rendering them feasible for application in young infants [31,40,41,42].

In this institution, the choice between the two procedures is largely based on a given family’s preference, which is itself only possible following a shared decision-making process that weighs the balance between the benefits and risks of the respective procedures. In the transcatheter group, elective intubation was not needed. For symptomatic infants dependent on oxygen or CPAP, transcatheter intervention seemed more attractive. This may result in an older population and a larger body weight in the baseline character of the transcatheter group.

Comparing surgical ligation with transcatheter closure is an emerging research area in the field of preterm infant care. Kim et al. reported no procedure-related mortalities or deaths within 30 days, but they did find that more inotropic agents support patients in the surgery group [33]. Pamukcu et al. conducted a comparison study between transcatheter and surgical PDA closure [31], and they demonstrated similar complications in the transcatheter and surgical ligation groups.

Ogando et al. also noticed that the surgical ligation group exhibited higher rates for intraventricular hemorrhage and inotropic therapy than the transcatheter group. The transcatheter group exhibited lower hospital mortality rates before 36 weeks of age [32]. In our limited study, the incidence of prematurity-related complications failed to demonstrate differences between the two procedures. To date, surgical ligation and transcatheter closure can be considered as therapy for preterm infants with cardiopulmonary compromise when medical treatment fails [18].

A large-numbered study conducted by Sathanandam reported that the time it takes for the respiratory severity score (RSS) to return to baseline is shorter in the transcatheter group as well as a faster weaning of invasive ventilator in [30]. In our study, we noticed a similar result in respiratory trajectory using a pulmonary score, which possibly reinforces more effective respiratory improvements. However, we failed to demonstrate differences in intubation days, which was probably due to the limitation of our small case numbers.

### 4.2. Respiratory Outcomes after Invasive PDA Closure

Invasive interventions to improve the respiratory condition in VLBW infants with a refractory PDA were reported and suggested in the 1970s and 1980s [20,43,44,45,46]. Ravel et al. first described the detailed respiratory outcome of 197 infants after surgical ligation in the 2000s; premature infants undergoing PDA ligation did not experience the anticipated rapid improvements in cardiorespiratory status [46]. Contrary to our expectations, pulmonary condition after invasive PDA management has been addressed in detail by a limited number of studies.

Ogando et al. have reported a pulmonary score difference between surgical ligation and transcatheter closure patient groups [32]. An increased score on Day 2 after surgical ligation was reported, and it failed to improve until Day 7; however, the score decreased immediately after transcatheter closure. We noted similar changes in our data. The pulmonary score increased immediately after surgical ligation, remaining high until Day 6. In the transcatheter occlusion group, the score also increased after the procedure but decreased on Day 2. In our data, the pulmonary score also decreased after the seventh day of surgery, and there was no statistical difference after Day 7 between the two procedures (Appendix A Table A2). This indicated that the respiratory condition of infants recovered slower in the surgical group, but long-term impact was not different compared with the transcatheter technique. Hsu et al. reported that high-frequency ventilation may be required for accelerating respiratory improvement after surgical ligation [47].

Recently, the important work of Sathanadam et al. demonstrated that compared to surgical ligation, catheter intervention may offer faster weaning of respiratory support when performed earlier in life and before the onset of elevated respiratory support [30]. Their well-designed study also demonstrated the feasibility and safety of catheter intervention in infants as small as 640–2000 g. In our observation of this study (Table 2), we found significantly fewer positive pressure ventilator-dependent days and invasive ventilator support days in infants receiving catheterization than in infants with surgical ligation. Our preliminary finding could be compared to the results of Sathanadam’s study.

### 4.3. Potential Benefits and Disadvantages for Transcatheter Occlusion

The several potential benefits of transcatheter occlusion should be addressed, as it is less complicated than surgical ligation. First, unilateral vocal cord paralysis following PDA surgical ligation has been frequently reported and associated with increased requirements for tube feeding, respiratory support, and a longer hospital stay [25,27,28]. Second, as a result of PDA ligation’s adverse impact on ventilator dependency and correlation to a higher incidence of severe bronchopulmonary dysplasia, infants receiving this treatment have been reported to suffer from diaphragmatic paralysis due to phrenic nerve injury. [48]. Third, cardiovascular instability after patent ductus arteriosus ligation (i.e., post-ligation cardiac syndrome, PLCS) in preterm infants has been reported [49], as it has a tendency toward a higher risk of long-term neurodevelopmental impairment [50].

Compared to infants with surgical ligation, VLBW infants who received transcatheter closure experienced less post-ligation cardiac syndrome and had less escalation of respiratory support [51]. This finding implies that PLCS may be related not only to the obliteration of the ductus but also to the procedure itself. In our series, all 31 patients reported no significant hemodynamic change between their conditions before and after the procedures. Thoracotomy during surgical ligation may cause atelectasis of the lung, iatrogenic pneumothorax, and impairment of the chest wall muscular function, which may not occur after transcatheter treatment.

Moreover, in our data, no patient required additional intubation for transcatheter occlusion. All infants intubated had pre-existing lung conditions. However, eight of those who received surgery required additional intubation. In our study, the feasibility of performing transcatheter occlusion without invasive ventilation prevented infants from unnecessary endotracheal tube intubation. This study provided encouraging preliminary data, and further investigation is needed to establish the correlation.

However, the transcatheter technique has been associated with some potential risks, including hypothermia, device migration, vascular injury, left pulmonary artery stenosis, and the coarctation of the aorta [39,52,53,54,55]. The one procedure-related mortality of this study was due to device migration, which was found 163 days after the procedure. Procedural charges have also been reported as being higher for transcatheter closure, which is driven by device charge and catheterization room utilization [33].

### 4.4. Limitations and Strengths

This study has some major limitations. Firstly, it was a retrospective study. The assignment was largely based on the choice made by a given patient’s family after the benefits and risks were clearly explained by an interventionalist and surgeon, respectively. Although baseline demographic data and medical condition were statistically similar, Group A seemed to include older GA infants, which can make a difference in post-op respiratory outcomes. The population was small, and the values of our results varied considerably.

However, a tendency toward earlier improvement in the respiratory trajectory could still be observed, as was the case in other studies [30,32]. Our observation is worthy of more extensive research in the future, with larger sample sizes and longer observation.

## 5. Conclusions

In this small case study of VLBW infants with hemodynamic-significant PDA, we observed a clear improvement in respiratory score from Day 3 to Day 5 after transcatheter closure. However, respiratory outcomes are not statistically significant between the two interventional techniques, despite them showing a trend toward early improvement in post-intervention respiratory trajectory in the transcatheter group. Future research with larger case numbers should be conducted to explore our observations in more detail.

## Figures and Tables

**Table 1:** Baseline characteristics of the study groups.

	Transcatheter Occlusion	Surgical Ligation	*p*-Value
**Patient information**	*n* = 14	*n* = 17	
Sex, *n* (male: female)	5:9	9:8	0.337
Birth gestational age, weeks	25.8 (23–29.4)	24.7 (23.6–25.4)	0.360
Birth BW, grams	772.5 (555–1330)	731 (684–744)	0.634
**Data related to intervention**			
Age of procedure, days	20.5 (12–35)	26 (18–38)	0.439
median (IQR)			
Age range (days)	2–91	11–47	
D0 Body weight, grams	1278 (478–1602)	795 (551–1646)	0.284
median (range)			
PMA on procedure day (weeks)	30.4 ± 3.8	28.7 ± 1.9	0.149
**Hemodynamics before procedure**			
HR (beats per minutes)	161 ± 15	158 ± 9	0.514
MBP (mmHg)	47.2 ± 13.4	43.6 ± 10.5	0.397
**Pre-procedural ventilation status**			
FiO_2_ (%)	33.4 ± 14.2	31.1 ± 8.8	0.573
PEEP (cmH_2_O)	5 (5–6)	5 (5–5.5)	0.902
Mean airway pressure (cmH_2_O)	11 (8.0–16.3)	8.65 (7.9–10.3)	0.082
Invasive ventilation, (*n*, %)	8/14 (57%)	9/17 (52%)	1.000
IMV, *n*	2	8	
NAVA, *n*	0	1	
HFOV, *n*	6	0	
Non-invasive ventilation, (*n*, %)	6/14 (43%)	8/17 (48%)	
CPAP, *n*	4	3	
NIPPV, *n*	2	3	
NIV NAVA, *n*	0	2	
**Medications before procedure**			
Methylxanthines, *n*	8	12	
Diuretics, *n*	3	3	
Postnatal steroids, *n*	3	2	
Pre-procedural pulmonary score	0.66 (0.55–0.88)	0.63 (0.50–1.00)	0.705

Data are presented in mean ± standard deviation, median (interquartile range) (IQR), or ratio (%), if not specifically mentioned. Abbreviations: BW: body weight; FiO_2_ was expressed as a percentage; HR: heart rate; MBP: mean blood pressure; PEEP: positive end-expiratory pressure; PMA: post-menstrual age; IMV: intermittent mandatory ventilation; NAVA: neurally adjusted ventilatory assist; HFOV: high-frequency oscillatory ventilation; CPAP: continuous positive airway pressure; NIPPV: noninvasive intermittent positive pressure ventilation; NIV NAVA: noninvasive neurally adjusted ventilatory assist.

**Table 2:** Other respiratory outcomes after invasive intervention.

	Transcatheter Occlusion *n* = 14	Surgical Ligation *n* = 17	*p*-Value
Duration on invasive ventilator after procedure, days	4 (1.5–6.5)	4 (3–6)	0.654
Duration on positive pressure ventilator after procedure, days	31 (24–45.5)	67 (52.5–86)	0.033
Oxygen dependent days after procedure	38 (9–66)	59 (25.5–163)	0.083

The variables are presented in median [interquartile range] and analyzed with Mann–Whitney U test.

## Data Availability

The datasets used during the current study are available from the corresponding author on reasonable request.

## Appendix A

**Table A1:** Reasons for intervention.

	Transcatheter Occlusion (*n* = 14)	Surgical Ligation (*n* = 17)	*p*-Value
Bleeding tendency, *n*	5	3	0.245
Pulmonary hemorrhage, *n*	5	1	
Coagulopathy/Thrombocytopenia, *n*	0	1	
GI bleeding, *n*	0	1	
Failed medical treatment, *n*	6	13	
Necrotizing enterocolitis, *n*	1	0	
Renal failure, *n*	2	1	

Bleeding tendency includes coagulopathy, thrombocytopenia, pulmonary hemorrhage, and GI bleeding.

**Table A2:** Pulmonary score before and after invasive intervention.

	D-1	D0	D1	D2	D3	D4	D5	D6	D7	D8	D9	D10
Group A (*n* = 14)	0.80	1.01	0.82	0.70	0.55	0.57	0.57	0.49	0.58	0.54	0.48	0.46
	(0.11)	(0.17)	(0.16)	(0.12)	(0.07)	(0.07)	(0.07)	(0.04)	(0.10)	(0.07)	(0.06)	(0.04)
Group B (*n* = 17)	0.71	1.41	1.03	0.93	0.89	0.77	0.78	0.75	0.69	0.63	0.62	0.64
	(0.07)	(0.16)	(0.12)	(0.09)	(0.11)	(0.08)	(0.09)	(0.09)	(0.09)	(0.08)	(0.08)	(0.09)
*p*-value	0.504	0.097	0.296	0.072	**0.014**	**0.037**	**0.047**	**0.011**	0.285	0.241	0.102	0.052

Values are represented as mean (SEM); the patient numbers of Group A decreased to 13 after D1; *p* < 0.05 in bold.

**Table A3:** Mean blood pressure and inotropic agents.

	Transcatheter	Surgery	*p*-Value
**Mean arterial pressure, mmHg**			
Pre-procedural	39.0 ± 10.0	43.5 ± 10.5	0.238
Post-procedural	47.4 ± 13.1	43.8 ± 11.4	0.429
**Inotropic equivalent**			
Pre-procedural	5.0 ± 6.0	2.8 ± 5.6	0.309
Post-procedural	5.7 ± 6.6	3.9 ± 6.3	0.434

Pre-procedural arterial pressure was recorded 2 h before start of intervention. Post-procedural arterial pressure was recorded 2 h after the procedure. Inotropic equivalent (IE) was defined as: dobutamine × 1+ dopamine × 1 +milrinone × 15 + epinephrine × 100 (expressed in µg/kg/min). Pre-procedural IE was recorded before the start of procedure. Post-procedural IE was recorded as the highest value within 12 h after the procedure.

**Table A4:** Morbidities related to prematurity.

	Transcatheter	Surgery	*p*-Value
NEC	2	1	0.576
IVH	2	4	0.664
BPD	13	16	1.000
ROP	6	12	0.119

NEC: necrotizing enteropathy; IVH: intraventricular hemorrhage; BPD: bronchopulmonary dysplasia; ROP: retinopathy of prematurity.
